# The role of DEK protein in hepatocellular carcinoma for progression and prognosis

**DOI:** 10.12669/pjms.293.3345

**Published:** 2013

**Authors:** Li-juan Lin, Li-tian Chen

**Affiliations:** 1 Li-juan Lin, Department of Medical imaging, Eastern Liaoning University of Medicine, Dandong-city (118000), Liaoning- Province, P.R. China.; 2 Li-tian Chen, Department of Liver Transplantation Surgery, Xin Hua Hospital, Shanghai Jiao Tong University School of Medicine, Shanghai-city (200092), P.R. China.

**Keywords:** Carcinoma, DEK, Hepatocellular, Immubohistochemistry, Prognosis

## Abstract

***Objective: ***The study aim was to explore the role of DEK in tumor progression and prognostic of hepatocellular carcinoma (HCC).

***Methodology: ***DEK protein in 178 samples of HCC was evaluated by immunohistochemical method. Additionally, the correlation between DEK expression and the clinicopathological features was evaluated by x^2 ^test or Fisher’s exact test, the survival rates were calculated by the Kaplan-Meier method, and the relationship between prognostic factors and patient survival was also by the Cox analysis.

***Results: ***DEK protein expression was noted in 86 cases of HCC, and 61 cases of normal liver tissues. DEK positive rate were closely correlated with the tumor size, grade, AJCC stage and survival rate (P<0.05, respectively). HCC with large tumor, lower grade, and late-stage, concomitant with DEK expression, had the lowest 5-years survival rate than HCC with above factors but without DEK expression (P<0.01, respectively). DEK expression emerged as significant independent hazard factors for survival in HCC (P<0.01).

***Conclusions: ***DEK could promote aggressiveness of cancer behavior, and hence poor prognosis of the HCC. It might be an independent poor prognostic factor and can serve as a useful new therapeutic biomarker.

## INTRODUCTION

Hepatocellular carcinoma (HCC) is the most common primary hepatic malignancy, especially in Africa and Asia.^1^^,^^2^ Despite many years of sustained efforts, the long-term outcome employing current therapies remain dismal as both recurrences of the lesions within the liver and distant metastases continue to increase. Therefore, both understanding and identification of useful biomarker for disease progression are of central importance for optimizing treatment outcome.

The proto-oncogene DEK protein was originally identified as a fusion with the CAN/NUP214 nucleoporin in a subset of acute myeloid leukemia patients.^3^^,^^4^ In many human aggressive tumors such as neuroblastoma, bladder cancer, cervical cancer, melanoma and the acute leukemia of adults, DEK was subsequently reported as a gene that is frequently upregulated.^5^^-^^9^ The available evidence suggests that DEK protein is closely related to apoptosis by its ability to inhibit p53-mediated apoptosis, cooperate with the viral oncogenes E6 and E7 to overcome senescence, promote HRAS-driven keratinocyte transformation, and promote cellular motility and invasion.^4^^,^^10^^-^^12^

Previous studies^13^ have showed that DEK protein was closely related with the proliferation of serous ovarian tumor cell, and DEK overexpression was significantly correlated with the increased proliferating index of Ki-67 in cervical cancer. Furthermore, it was found that DEK expression is correlated with the prognosis of a variety of human tumors such as breast cancer and ovarian cancer, is expected to become the new molecular targets of the cancer treatment.^14^ However, there is no study which has addressed to support increased DEK protein levels in HCC patients. This study was conducted to investigate the relationship of DEK expression with clinical pathologic parameters and prognosis in HCC patients.

## METHODOLOGY

***Samples: ***All the 178 cases were routinely processed and diagnosed HCC with strict follow-up were randomly selected from the patients undergoing surgery between 2004 and 2007 in the Liaoning and Jilin regions of China. The pathological parameters, including age, gender, size, grade, the presence of cirrhosis and nodal metastasis, portal vein tumor thrombus (PVTT), clinical stage, and survival data were carefully reviewed in all above cases. The patients’ age ranged from 32 to 73 years with a mean age of 55.4yr. The male to female ratio was 116:62. Tumor was staged according to the 7th edition of the American Joint Committee on Cancer (AJCC).^15^ Of the 178 HCC encompassed 100 cases as early-stage (I-II) and 78 cases as late-stage (III-IV). In which, 38 cases as well differentiated and 140 cases as worse differentiated cancers (grade). All the adjacent benign liver tissue from cancer resection margin were also included, and none of these patients had received chemotherapy before surgery.

All samples were approved by the Institutional Review Board, and informed consent from all patients were obtained before sample collection.

***Immunohistochemistry for DEK in paraffin-embedded tissues: ***Tissue sections were deparaffinized, rehydrated and incubated with 3% H_2_O_2_ in methanol for 15 minutes at room temperature to eliminate endogenous peroxidase activity. The antigen was retrieved at 95°C for 20 minutes by placing the slides in 0.01 M sodium citrate buffer (pH6.0). The slides were then incubated with primary antibody DEK (1:50, BD Biosciences Pharmingen, CA, USA) at 4°C overnight. After incubation at room temperature for 30 minutes with biotinylated secondary antibody, the slides were incubated with streptavidin-peroxidase complex at room temperature for 30 minutes. Immunostaining was developed by using chromogen, 3,3′-diaminobenzidine and counterstained with Mayer’s hematoxylin. We used the Mouse IgG isotope controls, which showed negative staining. Also, the positive tissue sections were processed omitting the primary antibody (mouse anti-DEK) as negative controls.

The interpretation criteria were described previously.[6] Briefly, the immunostaining for DEK was semi-quantitatively scored as negative ("-", no or less than 5% positive cells), "+" (5~50% positive cells), and "++" (more than 50% positive cells was). Only the nuclear expression pattern was considered as positive staining which included the weakly(+) and strongly (++) positive cells.

***Follow-up observation: ***All 178 patients had been followed-up for 5 years or until death. At the end of follow-up, 94 patients remained alive.

***Statistical analysis: ***Statistical analyses were performed using the SPSS 17.0. Correlation between DEK expression and clinicopathological characteristics was evaluated by x^2 ^test and Fisher’s exact test. The survival rates after tumor removal were calculated by the Kaplan-Meier method, and difference in survival curves was analyzed by the log rank test. Multivariate survival analysis was performed on all the significant characteristics measured by univariate survival analysis through the Cox proportional hazard regression model. P<0.05 was considered as statistical significance.

## RESULTS

**Fig.1:**
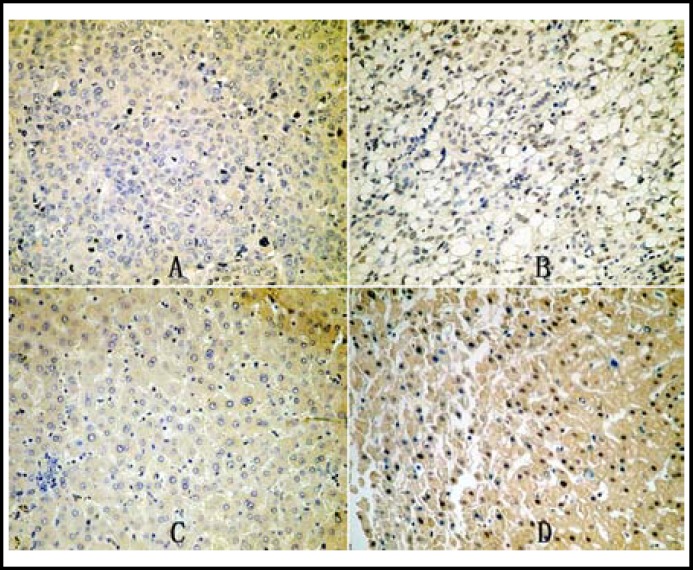
Immunohistochemical staining of DEK in HCC and benign liver tissue. DEK is negative (A) and positive (B) in HCC; DEK is negative (C) and positive (D) in benign liver tissue. (Original magnification, ×200

***Expression of DEK in HCC: ***DEK expression showed nucleus immunohistochemical staining pattern in HCC. There was statistical significance between the expression (+and ++) in the HCCs (48.31%, 86/178) and adjacent benign liver tissues (25.28%, 45/178) (P<0.001). (Fig.1)

***Clinicopathological and prognostic significance of DEK expression: ***To evaluate the role of DEK in HCC progression, we correlated DEK expression with major clinicopathological features, Table-I and Fig.3 shows that the DEK expression rate in the large tumors (>3cm), lower grade tumors (moderate of poor differentiation), and late-stage tumors (III-IV) was significantly higher than the smaller (≤3cm), higher grade (well differentiation), and early-stage (I-II) cases (P=0.023, 0.007, and 0.005, respectively). However, no statistical difference was found between DEK expression and age, gender, cirrhosis status, lymph node metastasis (LN), and PVTT (P>0.05, respectively).

***The correlation between the survival status and DEK expression by Kaplan-Meier*** ***method:***

**Fig.2:**
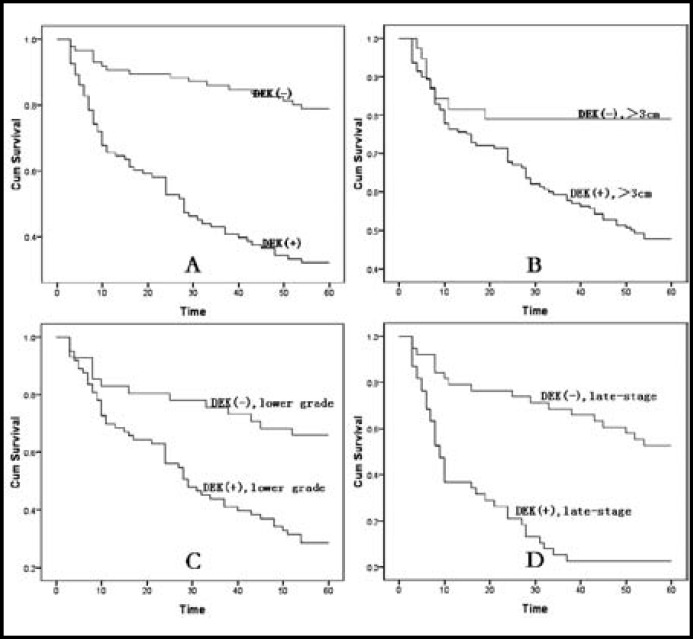
Kaplan-Meier analysis of 5-years survival in 178 HCC patients in relation to DEK protein expression. (2A) Patients with DEK expression had a significantly Lower 5-years survival rate (P<0.001). (2B) HCC with larger tumor concomitant with DEK expression were associated with the worst 5-years survival, significantly worse than HCC with larger tumor only (P=0.007). (2C) HCC with lower grade concomitant with DEK expression had the lower 5-years survival than HCC with lower grade only (P=0.001). (2D) HCC with later-stage concomitant with DEK expression were associated with the worst 5-years survival, significantly worse than HCC with later-stage only (P<0.001).

To further confirm the role of DEK expression in HCC progression, we analyzed the 5-years survival rate of 178 HCC cases by Kaplan-meier, and found that HCC patients with DEK expression had a lower 5-years survival rate than those without DEK expression, P<0.001 (Fig. 2A). Meanwhile, we also analyzed the correlation between other factors and the 5-years survival rate in HCC, and found that age, size, grade, PVTT, and AJCC stage were the key factors associated with 5-years survival rate. By combination analysis, we found that HCC with large tumor, lower grade, and tumor stage concomitant with DEK expression showed significantly lower 5-years survival rate than HCC with above factors but without DEK expression (Fig. 2B-D, P=0.007, P=0.001, and P<0.001, respectively), but no statistical relationship is seen between the case with and without LN and PVTT (P>0.05).

***DEK expression is an independent prognostic factor in HCC by Cox proportional hazard regression model: ***On univariate analysis all significant variables in Kaplan-Meier test, patients of HCC tumors with DEK expression had a significantly lower 5-years survival than those without DEK expression tumors (HR: 0.516, 95% CI: 0.383-0.694, P<0.001). Meanwhile, age, tumor size, tumor grade, LN, PVTT, and stage, were also associated with 5-years survival rate. Additionally, multivariate analysis was performed using the Cox proportional hazards model. We found that, larger tumor, lower grade, PVTT positive, and late-stage of HCC proved to be independent prognostic factors for survival in HCC. Importantly, DEK expression emerged as significant independent prognostic factors in HCC (HR: 0.161, 95%CI: 0.102-0.256, P<0.001) (Table-II).

## DISCUSSION

DEK is located on chromosome 6p22.3, initially described as the target of a recurrent t(6;9) translocation in a subset of acute myeloid leukemia (AML) patients. The human DEK protein consists of 375 amino acids with four distinct tretches of acidic amino acids. It has a central SAP box DNA-binding domainandan additional carboxy-terminal DNA-binding region that partially overlaps with a multimerization domain.^16^

**Table-I:** Relationship between DEK expression and clinicpathological factors

*Characteristic *		*Cases*		*Positive Cases *		* P value*	
Gender						0.521	
Male		116		54			
Female		62		32			
Age (Y)						0.426	
≥55		99		51			
<55		77		35			
Tumor size (cm)						0.023*	
≤3		67		25			
>3		111		61			
Tumor grade						0.007**	
higher		38		11			
lower		140		75			
Cirrhosis						0.587	
-		51		23			
+		127		63			
LN						0.159	
-		61		25			
+		117		61			
PVTT						0.113	
-		103		55			
+		75		31			
TNM						0.005**	
Early		102		40			
Late		76		46			

*P < 0.05,    **P< 0.01

**Table-II:** Univariate survival analyses (Cox regression model) of various factors in 178 patients

*Factors *	*B *	*SE *	*Wald *	*HR(95%CI) *	*P value*
Univariate analyses						
Age	0.333	0.151	4.844	1.396(1.037-1.878)	0.028*
Gender	0.007	0.155	0.003	0.987(0.737-1.355)	0.897
Tumor size	-0.304	-0.155	3.840	0.738(0.545-1.000)	0.049*
Tumor grade	-0.386	-0.157	6.077	0.680(0.500-0.924)	0.013*
Cirrhosis	0.003	0.167	0.000	1.003(0.723-1.390)	0.987	
LN	0.473	0.159	8.863	1.604(1.175-2.190)	0.003**
PVTT	-0.307	0.152	4.075	0.736(0.546-0.991)	0.043*
TNM	-0.763	0.154	24.498	0.466(0.345-0.631)	0.000**
DEK	-0.766	0.153	19.011	0.516(0.383-0.694)	0.000**
Multivariant analyses					
Age	0.258	0.157	2.687	1.294(0.951-1.762)	0.101
Tumor size	-1.192	0.243	24.022	0.304(0.188-0.489)	0.000**
Tumor grade	-0.323	0.169	3.643	0.727(0.519-1.009)	0.056	
LN	0.318	0.168	3.589	1.374(0.989-1.908)	0.058	
PVTT	-0.380	0.173	4.804	0.684(0.487-0.961)	0.028*	
TNM	-0.814	0.188	18.745	0.443(0.307-0.641)	0.000**	
DEK	-1.825	0.236	59.803	0.161(0.102-0.256)	0.000**	

*P < 0.05,    **P < 0.01

DEK protein function in the cells has not been clarified. Researches has shown that DEK is a phospho-protein with several phosphorylation sites of which most are clustered in the carboxy terminal region.^17^ The protein is localized strictly in the nucleus and occurs in copy numbers ranging from about one million copies/nucleus in proliferating lymphocytes to 4–6 million copies/nucleus in cultured HeLa cells. However, the abundance of DEK changes with the physiology of the cell, it is high in proliferating cells, and low in resting and terminally differentiated cells.^16^

Subsequently, DEK has been shown to promote tumorigenesis in a variety of cancer cell types, at least in part by its ability to interfere with cell division or DNA repair, inhibit cell differentiation, senescence and apoptosis, and cooperate with transforming oncogenes. Kavanaugh GM, et al.^18^ reported that DEK overexpression promotes the transformation of human keratinocytes, and that DEK knockout mice are partially resistant to chemically induced papilloma formation. Moreover, Trisha, et al^5^ used littermate DEK knockout, heterozygous and wild type mice for their experiments, and found that there was a significant delay in the formation of papillomas in DEK knockout mice compared to wild type and heterozygous mice. Papillomas ultimately formed in the DEK knockout mice, suggesting a role for DEK in tumor initiation in this model. Shibata, et al^19^ also showed that DEK overexpression, partly through an increase in its gene dose, mediates the activity of global transcriptional regulators and is associated with tumor initiation activity and poor prognosis in high-grade neuroendocrine carcinoma. Interestingly, some reports have indicated that DEK expression correlates with resistance to chemotherapeutic drugs like camptothecin, etoposide, neocarzinostatin, and doxorubicin, which is often used to treat breast cancer.^20^^-^^22^ In addition, recent reports have shown that DEK mRNA expression is up-regulated in invasive ductal breast cancers with particularly strong gene expression in high grade and late stage breast cancers, making it a potential new target in the fight against recurrence.^5^^,^^20^^,^^23^^,^^24^

At present, the reports of relationship of HCC and DEK has not been retrived yet. In this study, we performed immunohistochemical staining and analysis in 178 cases of HCC and their adjacent benign tissues, and found that DEK played important roles in the progression of HCC potentially. HCC with DEK expression correlated with large, high grade, late-stage tumors. Additionally, the combination of these indicators are more conductive to the HCC prognosis assessment. By combination analysis, DEK expression significantly affect 5-years survival rate of HCC, and the HCC with larger tumors, lower differentiation, and later-stage, concomitant with DEK expression, had the significantly lower 5-years survival rate than HCC with the factors but without DEK expression. Further analysis showed that DEK expression emerged as significantly independent hazard factors for survival in HCC. These findings led us to suggest that DEK promoted aggressiveness of cancer behavior, and hence poor prognosis of the HCC.

Our findings are perhaps the first to report on the prognostic significance of DEK in HCC, and proved DEK as a potential biomarker for HCC to evaluate its role in tumor progression and prognosis. DEK expression was more commonly seen in cases having poor prognostic factors of HCC, leading to bad differentiation, late-stage, and poor prognosis. DEK may serve as a useful new therapeutic biomarker. However, further studies are needed to confirm these observations.

## Authors Contributors

LLJ conceived the study. CLT performed research and wrote the first draft. All authors contributed to the design and interpretation of the study and approved the final version of the manuscript.
